# A review of neuroeconomic gameplay in psychiatric disorders

**DOI:** 10.1038/s41380-019-0405-5

**Published:** 2019-04-30

**Authors:** Siân E. Robson, Linda Repetto, Viktoria-Eleni Gountouna, Kristin K. Nicodemus

**Affiliations:** 1 Centre for Genomic and Experimental Medicine, Institute of Genetics and Molecular Medicine, University of Edinburgh, Edinburgh, UK; 2 Centre for Cognitive Ageing and Cognitive Epidemiology, University of Edinburgh, Edinburgh, UK

**Keywords:** Neuroscience, Psychology, Schizophrenia, Depression, Autism spectrum disorders

## Abstract

Abnormalities in social interaction are a common feature of several psychiatric disorders, aligning with the recent move towards using Research Domain Criteria (RDoC) to describe disorders in terms of observable behaviours rather than using specific diagnoses. Neuroeconomic games are an effective measure of social decision-making that can be adapted for use in neuroimaging, allowing investigation of the biological basis for behaviour. This review summarises findings of neuroeconomic gameplay studies in Axis 1 psychiatric disorders and advocates the use of these games as measures of the RDoC Affiliation and Attachment, Reward Responsiveness, Reward Learning and Reward Valuation constructs. Although research on neuroeconomic gameplay is in its infancy, consistencies have been observed across disorders, particularly in terms of impaired integration of social and cognitive information, avoidance of negative social interactions and reduced reward sensitivity, as well as a reduction in activity in brain regions associated with processing and responding to social information.

## Introduction

Psychiatric disorders are prevalent and often debilitating conditions for which the underlying biological causes or contributing factors are largely unknown. Research progress has been limited by a lack of measurable biomarkers to distinguish categories of disorders, relying instead upon self-report criteria. Here, we review how differences in social and cognitive processing measured through neuroeconomic gameplay may offer one useful approach towards the ambitions of the Research Domain Criteria (RDoC) initiative, proposed by the National Institute of Mental Health (USA), which advocates such a biomarker approach [1]. As we will describe, these are a set of easily applied games that show both shared and differential behavioural and brain responses across conventional diagnostic boundaries.

Social interaction is an intrinsic feature of human behaviour and any disruption to the ability to understand or act upon social information can have a serious impact on everyday life. Making decisions in social situations is a complicated process that requires substantial cognitive capacity as well as awareness of the context of the decision, inference of others’ emotion (theory of mind (ToM)), understanding the motivations for others’ actions and consideration of the potential consequences of decisions for both parties. At a neuronal level, these processes require rapid integration of complex information across a network of brain regions. It is therefore not surprising that social cognition is impaired in many, if not all psychiatric disorders [2], and is in fact one of the diagnostic criteria for some disorders (e.g., autism).

Functional neuroimaging techniques have shown that social information processing involves networks of multiple cortical brain regions, including areas associated with emotion and reward, such as the anterior insula, orbitofrontal cortex (OFC) and rostral anterior cingulate cortex (rACC); the perception and evaluation of social stimuli, including the medial prefrontal cortex (mPFC), temporal poles, superior temporal sulcus (STS), temporoparietal junction (TPJ), paracingulate cortex and precuneus; regulation of reactions to stimuli, including the dorsolateral prefrontal cortex (dlPFC) and dorsal ACC [2–4]. Disruption to these networks can result in impairments in social cognition, producing similar symptoms across different psychiatric disorders [5], although the neural mechanisms underlying the symptoms may or may not differ [2]. Thus, paradigms that measure these impairments across different diagnoses in the spirit of the RDoC framework will shed light on brain dysfunction across the spectrum of mental ill health.

Game Theory paradigms allow social decision-making to be studied experimentally by mimicking real-life social interactions in controlled laboratory settings, providing an intermediate step between social behaviour and the underlying neurobiological mechanisms [6]. These paradigms involve dynamic interactions between two or more players in strategic scenarios, and factors influencing players’ decisions can be tested against predictions from mathematical models of the mechanisms the games represent. These games are widely used in behavioural and psychiatric research because of their generalisability: they are effective tools for assessing prosocial and antisocial actions in healthy participants and can be used to evaluate abnormalities in social behaviour in clinical groups. We will focus on a subgroup of these Game Theory paradigms called neuroeconomic games, which combine economics, psychology and neuroscience into a general theory of human behaviour [7]. We will first describe each game and then outline behavioural and neuroimaging findings in clinical psychiatric populations.

## Neuroeconomic games

### Ultimatum Game

**Fig. 1:**
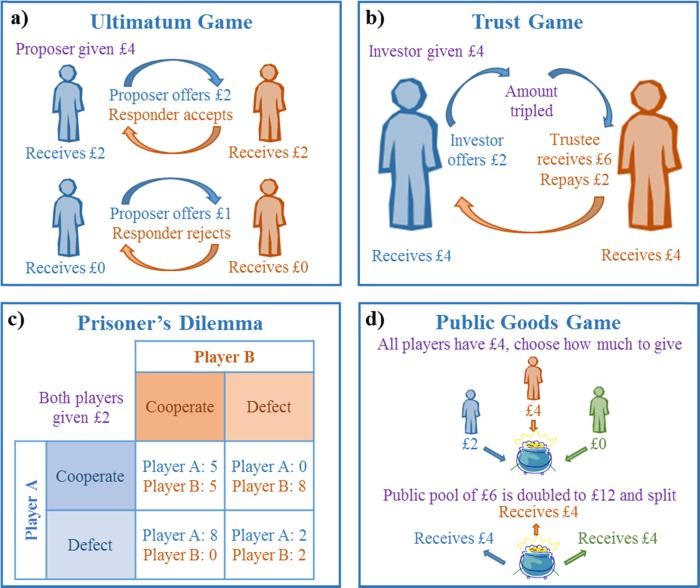
Schematic of the main neuroeconomic games. **a** Ultimatum Game, **b** Trust Game, **c** Prisoner’s Dilemma and **d** Public Goods Game

One of the simplest games, the Ultimatum Game [8] (Fig. 1a) evaluates players’ reactions to the fairness of offers of a share of money. In this game, the “Proposer” is endowed with a sum of money which they split between themselves and the “Responder”, choosing what proportion of the money to offer them. The Responder then decides whether to accept or reject the offer. If accepted, both players earn the amounts proposed, but if rejected, neither player earns anything. To maximise reward, the Proposer should offer the smallest possible share of the stack and the Responder should accept any offer larger than zero. However, Proposers tend to make “fair” offers of 30–50% of the stack [9], regardless of the stack size [10]. Fair offers are generally accepted, whereas unfair offers of less than about 30% tend to be rejected [9, 11]. When played in repeated rounds with the same partner (multi-shot format), the Responder’s acceptance rate typically influences the Proposer’s offers [5], so decisions are based on experience as well as the current situation.

Meta-analyses of functional magnetic resonance imaging (fMRI) studies have shown that when receiving unfair offers, brain areas related to emotion (insula, amygdala), cognition (PFC, ACC), reward processing (ventromedial PFC (vmPFC), striatum, posterior cingulate cortex (PCC) and putamen) and action planning (supplementary motor area (SMA)) show increases in the blood oxygen level-dependent (BOLD) response [11, 12]. These regions may form a reflexive emotional system (anterior insula and vmPFC) that is motivated to punish norm violations, and a deliberate system (ventrolateral PFC, dorsomedial PFC, dlPFC and rACC) that overrides the negative emotional response and suppresses self-interest to allow rejection of offers [12]. Receiving fair offers activates reward (vmPFC), emotion (posterior insula), visual (inferotemporal gyrus (ITG)), self-referential (precuneus) and empathetic (PCC) processing regions [12]. In Proposers making fair offers, both selfish and altruistic motives are evident from increased BOLD in areas related to reward (striatum and OFC), suggesting that they are thinking about their earnings, and areas related to ToM and morals (PFC, posterior parietal cortex (PCC)), showing that they are thinking about how the other player will feel [13]. When making unfair offers, there is an increase in the electroencephalography (EEG) medial frontal negativity response [14], which reflects an emotional reaction to violation of a social norm. This response indicates that participants have an aversive reaction to inequality despite its personal benefit.

### Dictator Game

The Dictator Game [15] is similar to the Ultimatum Game, except that the Responder has no choice but to accept the Proposer’s offer, regardless of the value, so it measures pure altruism from the Proposer. Offers tend to be lower than in the Ultimatum Game (around 20%), but depend on the context, e.g., knowledge of the characteristics of the Responder or the possibility of punishment for making unfair offers [9]. Making altruistic fair offers in this game is associated with moral decision-making and effort to overcome cognitive conflict, which have been associated with activity in the ACC, PCC, right supramarginal gyrus and right medial frontal gyrus [13, 16].

### Trust Game

Another well-established neuroeconomic game is the Trust Game [17], which measures both trust and trustworthiness or reciprocity (Fig. 1b). In this game, the “Investor” is endowed with a sum of money and can decide to send all, some or none of their endowment to the “Trustee.” Every unit is multiplied (usually by three) by the experimenter before reaching the Trustee, who decides whether to return all, some or none of the amount received to the Investor. The Investor could earn more by investing, but they risk losing out if the Trustee “defects” and keeps the money, rather than “reciprocating” and returning part of the multiplied investment. To maximise income, the Investor should not share any of their endowment and the Trustee should not return any of what they are given. However, almost all Investors send some money, generally around 50% of their endowment, and Trustees return approximately the amount the Investor sent to them [5, 9, 17, 18].

A review of the neuroscience underpinning the Trust Game proposed that different brain areas are involved at different phases: a cortical and subcortical network is implicated in decisions about trustworthiness; frontal areas are involved in deciding what to send/return; discovering the outcome activates reward circuitry, evaluation mechanisms and emotion-processing regions [19]. A meta-analysis of fMRI studies [20] reported that the anterior insula is active during decisions of whether to trust in single-shot games, suggesting an aversion to uncertainty. The ventral striatum response increases during multi-shot games, perhaps reflecting generation of predictions about outcomes and representations of the partner’s reputation. In Trustees, the decision of whether to reciprocate involves the anterior insula and intraparietal sulcus (IPS), indicating evaluation of options. During feedback about the Trustee’s response, increased activity has been observed in Investors’ dorsal striatum, suggesting reinforcement learning.

### Prisoner’s Dilemma

In the Prisoner’s Dilemma [21], individuals’ self-interest conflicts with that of the partnership, so it measures cooperation. Both players receive the same amount of money and simultaneously decide whether to cooperate (share) or defect (keep the money) (Fig. 1c). The total payoff is the greatest and is equally split if both cooperate; if both defect, the payoff is the lowest and equal, but a participant can earn the most if they defect and their partner cooperates. The optimal strategy is to always defect, but players cooperate around half of the time [18, 22], or more if communication is permitted [9]. Brain activity during this task occurs in areas involved in ToM (anterior paracingulate cortex and posterior STS), in encoding biographical memories (mid STS and hippocampus) and in emotional arousal (posterior cingulate and hypothalamus) [23].

### Public Goods Game

The Public Goods Game is similar to the Prisoner’s Dilemma, but is played in larger groups and it has a public and a personal pot. Earnings in the private pot are as specified, but earnings in the public pot are doubled and split between the participants (Fig. 1d). Again, earnings are maximised if all players cooperate, but a player will earn the most if they defect when all the others cooperate. The optimal strategy is to contribute nothing to the public pot, but people generally contribute around half to the public pot in one-shot games [22]. Cooperation declines over time unless there is a possibility for communication or punishment [5, 9].

### Aims

Since neuroeconomic games can provide an ecologically valid measure of social decision-making behaviour and its underlying brain activity, they might be used as a tool within the RDoC framework to assess abnormal function. Previous reviews of neuroeconomic gameplay in psychiatric disorders mostly describe individual disorders. This review therefore aims to summarise behavioural and neuroimaging findings from neuroeconomic gameplay studies of adults with Axis 1 psychiatric disorders, and to interpret the findings in the context of RDoC domains.

## Methods

The search for publications was conducted through the Medline (Pubmed), Web of Science and Scopus databases in September 2017. The search terms were (neuroeconomic* OR neuroeconomic* OR “economic game*” OR “trust game*” OR “ultimatum game*” OR “prisoner’s dilemma”) AND (psychiatr* OR psychotic OR psychosis OR psychopath* OR “mood disorder*” OR depressi* OR anxi* OR bipolar OR schizophren* OR schizotyp* OR “mental disorder*”). The date of the search was restricted to 1960 onwards. Only articles written in English were included, and only journal articles or books were included.

## Results

### Database searches

**Fig. 2:**
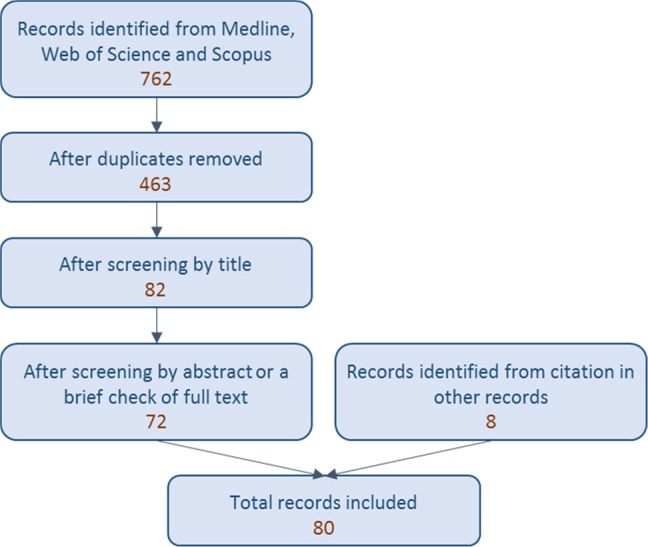
Summary of the search strategy and results

The search returned 762 records (Fig. 2): 222 from Pubmed, 216 from Scopus and 324 from Web of Science. After removal of duplicates, there were 463 records. These were assessed for relevance based on their title and a total of 82 were taken forward for review of the abstract or a brief look at the full text. Of these, 72 were considered to be relevant and the full text was reviewed. Eight additional records were included after identification through citations in articles returned by the search.

### Depression

This review will recap the main findings and add results of recent neuroeconomic studies of depression, as they have previously been reviewed [4, 24].

#### Ultimatum Game

When acting as the Proposer in the Ultimatum Game, patients with depressive disorders make more fair offers than controls [25] or say that they would offer more [26]. As the Responder, patients with depression reject more unfair offers than controls [26–28], as do healthy volunteers after induction of sad mood [29]. Rejection rate correlates with depressive symptom severity [26, 28] and does not change even after symptoms improve with therapy [26], implying that altered decision-making is a trait that manifests in patients with more severe depression and persists even after remission. Supporting the idea that increased rejection is mainly evident in more severe cases, studies reporting no difference between groups’ rejection rate of unfair offers [25, 30–32] or greater acceptance of unfair offers in depression [33] either involved nonclinical samples [31, 33] or patients with moderate depression [30, 32], and one used a paradigm in which the participant played both roles and so was motivated to accept offers in the hope that their subsequent offers would be accepted [25]. In a nonclinical sample, rejection rate for fair offers was not affected by depression [33]; however, in a clinical sample, rejection of both fair and hyperfair offers was greater in patients than controls [27], so depression seems to be associated with a general tendency to reject offers. Differences in patients’ performance on the Ultimatum Game may be due to several factors: avoidance of being rejected causing them to make higher offers [25]; reduced reward sensitivity causing greater rejection of offers [27]; pessimism and self-blame resulting in a focus on the negative associations of unfair offers rather than the potential benefit of accepting the offers [29, 32].

People with depression report more negative emotions than controls on receiving unfair offers, including guilt, disgust, surprise and anger [32, 33] as well as less happiness about fair offers [30]. They also find offers more unfair [27], although one study showed no difference in groups’ ratings of fairness [26]. The understanding of fairness is not affected since patients accept more fair than unfair offers [25] and the smallest share they regard as acceptable does not differ from controls [26]. Other decision-making processes are also intact: patients and controls react similarly to the Proposer’s emotion and to offers chosen over alterative available offers [27]. Interestingly, controls accept more very unfair offers of a small stake proposed by a computer rather than a human, but patients do not, suggesting that patients are only concerned with fairness and not with the social context of the offer [28].

In an fMRI study, although no differences in behavioural performance were observed, controls showed BOLD responses in the vmPFC, nucleus accumbens and dorsal caudate with increasing fairness, while patients only showed vmPFC activity [30]. The nucleus accumbens and dorsal caudate are part of the ventral and dorsal striatum, respectively, and the absence of response in these areas in patients may reflect abnormal processing of social fairness and reward. With increasing unfairness, both groups activated the dorsal ACC and insula, but only controls activated bilateral medial occipital cortex, which the authors propose reflects attentional disengagement from aversive social cues in patients. The BOLD responses to fairness in the nucleus accumbens and to unfairness in the left medial occipital region correlated negatively with anhedonia and symptom severity, suggesting that these blunted neural responses may contribute to patients’ negative experience of social interactions.

#### Trust Game

One study has reported no association between depression and performance as the Investor in the Trust Game, but levels of depression in the cohort were very low [31]. As the Responder in multi-shot Trust Games, patients with remitted major depressive disorder (MDD) show more reciprocity than healthy controls [34]. Another study found that depressed men showed more reciprocity than healthy men, but there was no difference for women; and suicidal ideation reduced self-centredness in men and increased it in women; so severe depression may reverse the typical profile whereby women are more prosocial than men [35]. The authors propose that behaving in a reciprocal way might help to reduce social stress. In Trust Games with a high risk of being caught “cheating” (returning less than the amount requested by the Investor), patients with MDD cheat less than controls, perhaps because reduced cognitive and affective processing limit the ability to deal with the cognitive load in this situation [36]. When the risk of detection is low, controls start to cheat but patients do not [37], and this behaviour is predicted by reductions in the BOLD response in the left dorsal putamen and anterior insula. These areas are involved in implementing action based on the risks and values of the situation, so that again they may reflect a reduced ability to deal with socio-cognitive demands of such situations [37]. If caught cheating, participants’ earnings would be confiscated, so the increased reciprocity and reduced cheating in patients with depression may also be due to a desire to avoid punishment.

#### Prisoner’s Dilemma and other cooperative games

People with depression are less cooperative in the Prisoner’s Dilemma game than healthy controls [32, 38] and have a more negative emotional reaction to betrayal: behaving more aggressively towards a betraying partner and being more critical of their own performance in another task after being betrayed [39]. They also express feelings of self-devaluation, which may reduce cooperation. Similarly, in healthy participants, depressive symptoms are associated with less cooperative behaviour on Prisoner’s Dilemma and Public Goods Games [31] or a reduced intention to cooperate [40]. However, one study reports that depressive symptoms in a nonclinical sample are associated with more sustained cooperation with a cooperative partner in a Prisoner’s Dilemma and with more changeable responses to an unbiased partner [41].

An fMRI study found no difference in behaviour of patients with MDD and controls, but while both groups showed BOLD responses in the anterior insula and dlPFC when one player reciprocated and the other defected, patients’ left dlPFC responses were reduced and this reduction correlated with guilt at not reciprocating cooperation [42]. The reduced dlPFC response may reflect difficulties with the higher cognitive demand of regulating emotions and making decisions during imbalanced social interactions. Patients were less satisfied with their earnings and reported more feelings of betrayal and guilt.

#### Summary: depression

Patients with depression have a more negative emotional reaction to unpleasant social interactions than healthy controls [30, 32, 39], and respond accordingly, e.g., rejecting unfair Ultimatum offers [26–28] and disengaging attention from the situation [30]. Although there is wider evidence for a negative emotional bias in depression, it is not clear whether this is a state or trait effect [43], so longitudinal studies of neuroeconomic gameplay could contribute to this debate in the context of unequal social interactions. The greater generosity [25] and reciprocity [34–37] observed in depression may be a mechanism to reduce the risk of negative social interactions, or may reflect guilt-based hyper-altruism [44]; however, evidence for reduced cooperation [32, 38] does not support this theory, so further research is required on the topic of prosocial decisions in neuroeconomic games in depression. People with MDD show a reduced ability to adapt their behaviour with changing circumstances in the games, indicating impaired integration of cognitive and affective information to inform decision-making. There is also behavioural [26–28] and neuroimaging [30] evidence for reduced reward sensitivity in depression. These behaviours all fall under the Affiliation and Attachment RDoC construct, which is part of the Systems for Social Processes domain; and decision-making and reward sensitivity fall under the Reward Valuation and Responsiveness constructs within the Positive Valence Systems domain, respectively. Some inconsistencies in the results across studies may be due to the differences in patient characteristics such as symptom severity and medication, and differences in the paradigms such as single- versus multi-shot [24, 45].

### Bipolar disorder

An Ultimatum Game study of patients in the euthymic phase of bipolar disorder found greater rejection of offers where fairness was ambiguous (around 30% of the stack) compared with controls, but similar acceptance of clearly fair and unfair offers [46]. Patients also made angry statements about the game, but some expressed regret at behaving impulsively and rejecting offers that lost them profit. In the Trust Game, patients with remitted bipolar disorder showed more reciprocity than controls, which is dysfunctional as it reduces personal gain [34]. Patients with bipolar disorder therefore behave similarly to people with depression in neuroeconomic games: reacting angrily to unfairness and showing increased reciprocity that could reflect altruism caused by feelings of guilt or avoidance of conflict.

### Anxiety disorders

#### Ultimatum Game

Generalised anxiety disorder (GAD) is associated with avoidance of social confrontation. One study has reported that patients with GAD accept more unfair Ultimatum Game offers and rate these as less unequal than both controls and patients with panic disorder [47]. Patients also reported no difference in emotional responses to fair and unfair offers, unlike controls. The authors propose that avoidance of conflict and a lack of anger at unfair treatment causes increased acceptance of unfair offers. Two studies have investigated Ultimatum Game behaviour in students with high- and low-trait anxiety (HTA/LTA) [48, 49]. As the Proposer, the groups made similar offers that were generally fair. As the Responder, when participants thought that they were interacting with a human, acceptance of unfair offers was negatively associated with self-esteem in HTA participants, but positively associated with impulsivity in LTA participants, perhaps reflecting avoidance of social exclusion and a desire to maximise profit regardless of fairness, respectively. The two studies found contradictory results in relation to acceptance of unfair offers from humans or computers in the two groups, indicating that the interaction between fairness, social context and anxiety requires further investigation. In parietal EEG electrodes, the P3 component involved in evaluating events was larger for human than computer proposers in the HTA but not the LTA group, so highly anxious people might find unfair offers from humans more salient than from computers, whereas less anxious people are less concerned about the social context [48]. The feedback-related negativity response in frontal electrodes was larger for unequal than equal offers in the HTA but not the LTA group, so highly anxious people may evaluate unfair offers more negatively, although this contradicts the self-reports of emotion in the study described above [47]. These suggest that high-trait anxiety is associated with similar avoidance of socially stressful situations as is seen in GAD; however, further studies are needed to show the relationship between clinically diagnosed anxiety and HTA.

#### Trust Game

As the Investor in a Trust Game, patients with generalised social anxiety disorder (GSAD) and controls are both more likely to cooperate with a cooperative Trustee, so there was no difference in behaviour between groups [50, 51]. However, there were differences in brain activity: in controls, a network of regions involved in social decision-making was active when playing a human as opposed to a computer partner; in patients, this network was less extensive and did not include the mPFC, an area involved in attributing mental states to others and in forming impressions about people [50]. Controls also engaged the ventral striatum during repeated exchanges with cooperative as opposed to neutral partners, while the GSAD group did not, and the severity of social anxiety symptoms predicted diminished responses to cooperative partners [51]. This finding could reflect hyperarousal during social interactions or a deficit in implicit learning of partners’ reputations and how to use this information for future decisions.

#### Prisoner’s Dilemma and other cooperative games

Cooperation may be more affected by other symptoms than by anxiety itself: patients with GSAD gave less than controls in a Prisoner’s Dilemma [52], but giving was more strongly associated with quality of friendships and the interpersonal traits of vindictiveness and coldness than by the diagnosis [52, 53]. In students with attachment anxiety, symptoms of anxiety and avoidance did not significantly predict cooperation, but highly anxious patients were slower to make decisions and less consistent in their performance on the Assurance Game but not the Prisoner’s Dilemma, indicating a chronic lack of trust, since the Assurance Game is a version of the Prisoner’s Dilemma that encourages cooperation by giving the highest payoff when both players cooperate. Priming patients with attachment security reduced this difference and increased cooperation on the Prisoner’s Dilemma, so trust in attachment anxiety can be increased by security of the social interaction [54].

Two studies on adolescents with anxiety disorders playing the Prisoner’s Dilemma report contradictory results: one found that after their partner cooperated, patients were more cooperative than controls, but there was no difference after defection [55]; the other found that patients were more cooperative after defection, despite feeling more negative about their partner, but there was no difference after cooperation [56]. The difference may be due to higher comorbidity of depressive disorders in the original study. Patients showed reduced BOLD activity compared with controls in the mPFC and ACC when discovering the partner’s response [56]. The reduced mPFC activity mirrors that observed in Trust Games [51] and could reflect a lack of engagement of prefrontal areas to monitor responses and integrate the decision-making brain network. The reduced ACC response may represent limited generation of expectations about partners’ behaviour. Patients also showed a greater BOLD response in the anterior precuneus and the right TPJ than controls [56], which could represent heightened focus on their own behaviour and rumination about what others are thinking, respectively.

#### Summary: anxiety

Patients with anxiety respond in the opposite way to those with depression in the Ultimatum Game, accepting more unfair offers and reacting less negatively to unfairness. Social decision-making in anxiety is influenced by internal factors such as personal characteristics, and by external influences like cooperation and perceived security, and is associated with abnormalities in prefrontal cortex activity.

### Schizophrenia spectrum and other psychotic disorders

The use of neuroeconomic games in schizophrenia and psychosis has been summarised in reviews focussed on social functioning [57] and moral cognition [58] that were published prior to several additional studies reviewed here.

#### Ultimatum and Dictator Games

As the Proposer in Ultimatum Games, patients with schizophrenia offer a greater share of the stack than controls, making more hyperfair and fair offers, and fewer unfair offers [59–61]. Similarly, in a nonclinical sample, only participants with high schizotypy scores made hyperfair offers in Ultimatum and Dictator Games, and higher offers were associated with greater positive (cognitive/perceptual and disorganised) symptoms [62]. As the Responder, patients with schizophrenia reject more offers than controls [59], or reject more fair and hyperfair offers [63, 64] but accept more unfair offers [63–65], as do students with schizotypal traits [62]. One study found the opposite: patients with schizophrenia rejected more unfair offers than controls [66] and another found no behavioural difference between patients and controls [61]. However, the bulk of the evidence suggests that individuals with schizophrenia or high levels of schizotypy behave less strategically on these games, failing to maximise profit [57, 58]. The greater acceptance of unfair offers may reflect reduced altruistic punishment in schizophrenia (punishing unfair behaviour at the expense of one’s own profit) [63], which may be due to patients’ tendency to choose imminent rewards rather than possible future gains [67]. The reason for increased rejection of fair and hyperfair offers could be impaired ToM, causing patients to be less aware of the generous intentions of the Proposer [64].

Acceptance of offers correlates with delusions and suspiciousness/persecution [63], and acceptance of unfair offers correlates with excitement and disorganisation [65]. These correlations with positive symptoms may be driven by patients being more apprehensive of the consequences of rejecting offers [63], or by increased feelings of victimisation [65], potentially resulting in an expectation of unfair treatment and so a less negative reaction to it. Rejection of fair offers correlates with negative symptoms [65], which we propose reflects low self-esteem, causing patients to believe that they are not worth the equal share.

Patients with schizophrenia also respond less consistently than controls, e.g., rejecting higher offers after accepting lower ones [63], and show less behavioural flexibility: failing to adapt to changes in the task or their partner’s behaviour. As the Proposer, they do not reduce their offer after acceptance of a previous offer [59], or offer lower amounts to a computer than a human partner [60] as controls do. Controls were also more likely to reject an unfair offer when the Proposer chose that over a fair alternative, whereas patients were not influenced by alternative offers [64]. ToM partially mediated this group difference, so patients may fail to see the alternative offer from the Proposer’s perspective. Smoking normalised this difference: patients who smoke showed similar reactions to alternative offers as controls and nonsmoking patients’ performance was improved by a 1mg dose of nicotine [68]. Patients are not affected by the Proposer’s emotion, while controls reject more unfair offers proposed with angry than other expressions [66] and accept higher offers if the Proposer’s expression was positive [63]. In a Dictator Game where participants could punish the Investor’s unfairness by giving to the Responder and taking from the Investor, patients showed similar likelihood and the amount of punishment to controls, suggesting that patients do not have the difficulty in recognising unfairness, and the degree of punishment was associated with depressive and negative symptoms [65], supporting findings from studies of depression that show a negative emotional reaction to unfairness.

In an EEG study, no difference was observed between patients with schizophrenia and controls playing the Proposer, but the amplitude of feedback-related negativity (FRN) in the dlPFC and mPFC was reduced in patients playing the Responder, which may reflect difficulty in interpreting others’ behaviours [61]. When anticipating the Responder’s decision, alpha oscillations in the frontal and temporoparietal regions measured using EEG correlate with the risk of rejection by human partners in controls, but by computer partners in patients with schizophrenia [60]. This activity correlated with positive symptoms and may reflect a lack of mentalising ability in patients, or a misattribution of salience to computer partners.

#### Trust Game

Several studies by the same research group have found that patients with early and chronic psychosis and their first-degree relatives have lower initial levels of trust than controls when playing the Investor in Trust Games [69–71], suggesting a potential genetic underpinning to the strategies employed, though one study found no difference in siblings’ cooperative behaviour [72]. Reduced trust correlated with negative symptoms in patients with early-stage psychosis, so that it could reflect a lack of social motivation [70]. Over time, these patients begin to trust cooperative partners, reaching similar levels of cooperation to controls, but their trust in deceptive partners did not drop as much as controls’, suggesting a limited reaction to violations of trust or reduced behavioural flexibility after negative feedback [70]. Patients with chronic psychosis invested less overall in a cooperative partner than controls, but groups did not differ when playing a deceptive partner [71]. When given prior information of a Trustee’s trustworthiness, controls and first-degree relatives increased their trust, but patients with chronic psychosis did not [69], again indicating less strategic decision-making and reduced behavioural flexibility. Patients with chronic psychosis show a smaller BOLD response than controls in the right TPJ when the Trustee responds, and a smaller signal in the right caudate nucleus during cooperative responses, which may indicate that patients find positive interactions less rewarding [71]. The reduced caudate signal also correlated negatively with patients’ paranoia scores [71]. Siblings of patients with psychosis also showed reduced activation of the right caudate and putamen during investments, and of the left insula during repayments [72], indicating that the aberrant functioning in reward-processing regions observed in psychosis may have biological origins that are shared in siblings.

#### Prisoner’s Dilemma and other cooperative games

In a Prisoner’s Dilemma where money could be lost as well as won, controls behaved less cooperatively, showing loss aversion. Patients with schizophrenia did not show this effect, but those with less severe symptoms behaved more like controls [73]. Patients were also more cooperative on a Public Goods Game than controls, but again showed less of an impact of changes in the game: only controls defected less when cooperation was enforced and the risk of losing money was removed, and controls were influenced by failure to earn a bonus on the preceding trial, while patients were not, suggesting that patients do not recognise the impact of the previous trial on subsequent trials [74]. The increased cooperation in patients comes at the expense of personal profit and may be due to reduced loss aversion, impaired ToM and poor integration of cognitive and affective information [74].

#### Summary: schizophrenia and psychotic disorders

In general, patients with schizophrenia and psychotic disorders behave less “strategically”, making higher offers in Ultimatum and Dictator Games [59–61], accepting more unfair offers [63–65] and rejecting more fair and hyperfair offers [63, 64]. They also show less trust [69–71] but more cooperation [73, 74] than controls, and are less flexible: failing to adapt to the availability of contextual information or to their partner’s emotion or performance [59, 60, 63, 64, 66, 69, 74]. Several reasons for these behaviours have been proposed, including risk avoidance, limited loss aversion, preference for short-term gain, feelings of victimisation, poor ToM, reduced social motivation and poor integration of cognitive and affective information. Behavioural performance and brain activity correlate with positive, negative and overall symptom severity, so social decisions are more affected in patients who are more unwell. It would be helpful for further gameplay studies to investigate early, chronic and relatives of people with schizophrenia/psychosis using the same tasks and ideally also to examine genetic markers of behaviour on the games.

### Autism spectrum disorders

One study has reported that children with ASD accept more unfair initial Ultimatum Game offers and reject more fair offers, perhaps because they do not recognise the other’s unfair or generous intent [75]. Interestingly, several studies have shown no abnormalities in neuroeconomic gameplay behaviour in people with ASD: performance was similar to controls when acting as the Proposer in the Ultimatum and Dictator Games [75], and in the Prisoner’s Dilemma [75–77], Trust Game [78] and Beauty Contest Game (where individuals must guess what others are thinking) [79]. However, there is some evidence that symptoms of ASD relate to performance on these games, again supporting the use of RDoC criteria to describe psychiatric disorders in terms of observable behaviours rather than diagnoses. Participants who failed ToM or mentalising tasks showed less cooperation in the Prisoner’s Dilemma [75, 76] and behaved less strategically: failing to exploit cooperation and reciprocate defection in the Prisoner’s Dilemma and making lower Ultimatum proposals [75]. Similarly, adults with more severe symptoms of ASD were more likely to follow a fixed strategy, whereas controls were more likely to consider their partner’s move and use ToM to guide their decisions in a Stag Hunt Game, which measures cooperation [80]. Patients with ASD have a severely diminished middle cingulate response compared with controls playing the Trustee in a Trust Game, which may represent a reduced representation of the social intent of their actions and could lead to a reduced ability to model the intentions of others [78]. Children with ASD also showed reduced BOLD responses in the left insula, TPJ and bilateral caudate during defection of a human partner in a Prisoner’s Dilemma, and less activity than controls in the right insula during defection of a computer partner [77], suggesting reduced engagement of a social salience network. Overall, patients with ASD do not behave differently to controls on neuroeconomic games, but reduced ToM and mentalising ability is associated with reduced cooperation and strategic performance, and patients show decreased recruitment of brain regions involved in social processing during the interactions. However, it should be noted that in two of these studies [75, 77], groups were not matched for intelligence, so future studies should clarify the contributions of ToM, mentalising and intelligence on gameplay performance in all participant groups, but particularly in ASD.

### Other disorders

#### Attention-deficit hyperactivity disorder

Children and adolescents with ADHD did not differ from controls in offers made as the Proposer in the Ultimatum Game, but patients made fewer fair offers in a Dictator Game [81]. Self-reports confirmed that the ADHD groups were able to take another’s perspective and show empathic concern, but they chose to make decisions based on strategy rather than fairness. A discussion article proposed that three networks are involved in neuroeconomic decisions in ADHD: the default mode network linking medial, prefrontal and posterior cingulate cortex, which alters understanding of utility, anticipation of outcomes, setting of goals and implementing aims; a dorsal frontostriatal network that affects executive function and decision-making; dopaminergic dysregulation of a ventral frontostriatal network that disrupts evaluation of future utility, feedback on outcomes and learning of associations between cues and outcomes [82].

#### Eating disorders

Women with and recovering from anorexia showed less reciprocity than controls as the Trustee in a Trust Game, and had a diminished precuneus and right angular gyrus BOLD responses to high offers by the Proposer compared with controls [83]. The degree to which they attribute positive experiences to other people was inversely correlated with activity in a social network, including the precuneus, so patients may have difficulty in recognising kindness. Responses to low offers were lower in the left fusiform area only in currently ill participants, so recovery might be linked to recognising malevolence.

#### Post-traumatic stress disorder

In a Trust Game and a non-social task with a similar format, women with assault-related PTSD were slower to learn the probability of success for decisions, and errors were less likely to affect their future decisions, suggesting that they are less flexible in using their experiences to guide future decision-making [84]. Patients were less trusting than controls after Trustees behaved uncooperatively and were less likely to return to initial levels of trust. Abnormal activity in patients’ TPJ during social prediction errors may represent overthinking of others’ intentions.

## Discussion

### Neuroeconomic games within the RDoC framework

Neuroeconomic games provide a “snapshot” of social functioning that is more ecologically valid than current diagnostic measures, such as interviews or self-report questionnaires. This type of measure aligns with the recent move towards using the RDoC framework to describe mental health in terms of behaviours and functions rather than diagnoses [1], an approach which is gathering momentum and potentially merits wider adoption. All neuroeconomic games measure social processing that falls under the Affiliation and Attachment construct in the Systems for Social Processes RDoC domain; and reward processing, which is reflected in the Positive Valence Systems domain. Table 1 shows the RDoC constructs that neuroeconomic games apply to, and through which dysfunctional social decision-making could be measured. The specific game to use will depend on behaviour of interest: e.g., fairness, trust, reciprocity and cooperation.

**Table 1:** RDoC domains and constructs that are involved in neuroeconomic gameplay

Domain	Construct	Process involved
Positive Valence Systems	Reward Responsiveness (RR)	Responses to possible, received and repeated reward
	Reward Learning (RL)	Predicting a positive outcome, modifying behaviour based on outcome
	Reward Valuation (RV)	Computing the probability and benefits of an outcome
Systems for Social Processes	Affiliation and Attachment (AA)	Processing social cues, social learning and forming relationships

### Synthesis of the literature review

Performance in neuroeconomic games differs between individuals with psychiatric disorders and healthy controls in several ways, with some similarities and some differences between diagnoses. Table 2 summarises the main results reviewed here and shows which RDoC construct each observed dysfunction applies to. There are two areas in which there is consistency across diagnoses: (1) impaired ToM and integration of social and cognitive processes, which result in less effective and flexible decision-making. These impairments fall under the Affiliation and Attachment, Reward Valuation and Reward Learning RDoC constructs. Examples of these behaviours include a reduced ability to process social information, react to changes in the task or make strategic decisions to optimise outcomes in schizophrenia, PTSD and ASD.

**Table 2:** Summary of the results of neuroeconomic gameplay studies in Axis 1 psychiatric disorders, the dysfunction implicated by the results and the RDoC construct associated with the dysfunction

Disorder	Game	Result	Dysfunction implicated	RDoC
MDD	Ultimatum/Dictator	Make more fair offers [25]	Avoidance of risk (of rejection)	AA/RV
			Hyper-altruism	AA
		Reject more offers especially unfair [26–28]	Aversion to negative interaction	AA
			Reduced reward sensitivity	RR
		React negatively to unfairness [30, 32]	Aversion to negative interaction	AA
		No change with computer vs. human [28]	Poor social/cognitive integration	AA/RV
		No BOLD in nucleus accumbens and dorsal caudate for fairer offers [30]	Decreased processing of fairness	AA
			Reduced reward sensitivity	RR
		Less occipital BOLD for unfair offers [30]	Attentional disengagement	AA
	Trust	More reciprocity [34, 35]	Avoidance of risk (of social stress)	AA/RV
			Hyper-altruism	AA
		Less cheating in low-risk situation [36, 37]	Poor social/cognitive integration	AA/RV
		Less BOLD in dorsal putamen, AI and DLPFC during low-risk cheating [37]	Poor social/cognitive integration	AA/RV
	Prisoner’s Dilemma/Public Goods	Less cooperation [32, 38]	Reduced altruism	AA
		More negative about betrayal [39]	Aversion to negative interaction	AA
Bipolar disorder	Ultimatum/Dictator	Reject more moderately unfair offers [46]	Aversion to negative interaction	AA
			Reduced reward sensitivity	RR
		React negatively to the game [46]	Aversion to negative interaction	AA
	Trust	More reciprocity [34]	Hyper-altruism	AA
	Prisoner’s/Public Goods			
Anxiety	Ultimatum/Dictator	Accept more unfair offers [47]	Avoid conflict	AA
		Report unfair offers as less unequal [47]	Less angry about negative interaction	AA
	Trust	No difference in reciprocity [50, 51]		
		Reduced BOLD in mPFC with human vs. computer partner [50]	Poor ToM and impression formation	AA
		Reduced BOLD in ventral striatum with cooperative vs. neutral partner [51]	Poor social/cognitive integration	AA/RV
	Prisoner’s Dilemma/Public Goods	Inconsistent results [52, 55, 56]		
		Reduced BOLD in ACC and mPFC to partner’s response [56]	Poor social/cognitive integration	AA/RV
		Increased BOLD in precuneus and TPJ to partner’s response [56]	Heightened self-focus and rumination on others’ behaviour	AA
Schizophrenia/psychosis	Ultimatum/Dictator	Make more fair or hyperfair and fewer unfair offers [59–61]	Avoidance of risk (rejection)	AA/RV
			Hyper-altruism	AA
		Accept more unfair [63–65], reject more fair and hyperfair [63, 64], but inconsistent results [61, 66]	Reduced altruistic punishment, poor ToM, victimisation and impulsivity	AA
		No change in altered situation [59, 60, 63, 64, 66]	Less flexibility, poor strategising	RL
		Reduced FRN in dlPFC and mPFC [61]	Poor ToM	AA
		Frontal and TPJ alpha when playing computer vs. human [60]	Poor mentalising, misattribution of salience to a computer	AA
	Trust Game	Less trust [69–71]	Low social motivation	AA
		No change in altered situation [69]	Less flexibility, poor strategising	RL
		Reduced BOLD in right TPJ and right caudate [71] during partner’s response	Reduced reward sensitivity	RR
	Prisoner’s Dilemma/Public Goods	More cooperation [73, 74]	Lack of loss aversion, poor ToM and poor social/cognitive integration	AA/RV
		No change in altered situation [74]	Less flexibility, poor strategising	RL
ASD	Ultimatum/Dictator	Accept more unfair, reject more fair [75]	Poor ToM	AA
	Trust	No group difference [78]		
		Less BOLD in middle cingulate [78]	Reduced processing of social intent	AA
	Prisoner’s Dilemma/Public Goods	No group difference [75–77]		
		Less BOLD in insula, TPJ and caudate [77]	Reduced social processing	AA

(2) Increased risk avoidance (of negative social interactions) and reduced reward sensitivity, which result in reduced profit-seeking. These behaviours are associated with the Affiliation and Attachment, Reward Valuation and Reward Responsivity RDoC constructs. Examples of avoidance of the risk of negative social interactions include increased generosity in depression and schizophrenia; tolerance of unfairness in anxiety, schizophrenia and ASD; reciprocity in depression and bipolar disorder. Examples of reduced reward sensitivity include rejection of possible reward in depression, bipolar disorder, schizophrenia and ASD and a reduced striatal response in depression.

There are two areas that show different effects across diagnoses, both of which fall under the Affiliation and Attachment RDoC construct: (1) the emotional reaction to negative interactions is more negative in MDD and bipolar disorder, but less negative in anxiety, and (2) there is mixed evidence for cooperative and altruistic behaviour. There are reduced levels of cooperation in depression: trust in schizophrenia and PTSD; altruism in ADHD; reciprocity in anorexia. However, there are increased levels of generosity in depression and schizophrenia; reciprocity in depression and bipolar disorder; cooperation in schizophrenia.

There has not been sufficient neuroimaging research to discriminate which brain regions are dysfunctional during all of the behaviours observed in neuroeconomic games; however, several regions involved in social, emotional and cognitive processing show differences between patients and controls, including the precuneus, caudate, cingulate cortex, insula, TPJ, mPFC and dlPFC. Most of these regions show reduced activity in patients, so they may reflect decreased sensitivity to the information required to play the games, or reduced communication and integration of information across social decision-making networks. Several studies have also reported differences in neural responses but no difference in behavioural performance, so patients could have underlying biological deficits that are obscured by compensatory behavioural strategies.

### Caveats

If neuroeconomic games are to be used in identifying deficits in social function in mental ill health, it will be necessary to ensure that more consistent procedures are followed: parameters such as the proportions of the stack available, knowledge about the identity or habits of the opponents, inclusion of a computer partner “control” condition and opportunities for cheating or punishing should be standardised in order to create a profile of the expected results in healthy controls, so deviations from these can be readily identified and attributed to a deficit in a particular function or neural circuit. Indeed, the sometimes-conflicting results reported here may be attributed to these differences as well as to moderate sample sizes, focus on clinical populations with varying degrees of severity and inclusion of nonclinical populations [24]. Neuroeconomic games are relatively complex tasks and there is evidence that memory and other cognitive functions might affect decision-making [67] and that social cognitive impairment overlaps with general cognitive impairment [69], so group comparisons should take into account intelligence and memory performance.

### Further directions

In addition to potentially being incorporated into the RDoC framework, future prospects for use of neuroeconomic games in psychiatric disorders could include use in neuroimaging studies of structural and functional connectivity of brain networks involved in social processing. Identification of abnormalities in this way could potentially lead to the ability to stratify patients into diagnosis-spanning subgroups.

Investigation of genetic factors associated with suboptimal performance on the games could lead to the identification of molecular processes that could elucidate molecular mechanisms and potentially druggable targets. There is some evidence that behaviour in neuroeconomic games is heritable, suggesting a genetic component to social decision-making. In the Trust Game, trust from the Investor shows heritability of 20% in a Swedish sample and 10% in an American sample, and trustworthiness of the Responder shows heritability of 18 and 17% in the two groups [85]. In the Ultimatum Game, over 40% of variation in rejection behaviour is explained by additive genetic effects [86]. In addition, studies in first-degree relatives have also shown a genetic component, which suggests that neuroeconomic gameplay may be useful as an observable behavioural endophenotype for genetic studies of psychiatric disorders [69–71]. Studies of the molecular genetics underlying neuroeconomic gameplay strategies have thus far been limited to candidate genes. More altruistic behaviour during the Dictator Game was observed in individuals carrying the RS3 long promoter region repeat of arginine vasopressin 1a (*AVPR1A*) versus those carrying the shorter repeat [87]. *AVPR1A* has long been known to be critical for social cognition and behaviour in both lower mammals and humans [88]. A variable number of tandem repeat (VNTR) functional polymorphism in the dopamine receptor D_4_ (*DRD4*) gene has been associated with fairness in the Ultimatum Game Responder role in a Chinese sample, where individuals with the 4/4 genotype (versus 2 allele carriers) required a greater threshold for the minimal acceptable offer, but there was no effect on the Proposer [89]. The effect of this polymorphism on the Responder and the absence of an effect on the Proposer have been replicated in an independent sample of individuals from Germany [90]. In addition, the German study reported an association between a haplotype block containing two single nucleotides in the dopamine receptor D_2_ gene (*DRD2*) and the behaviour of the Proposer, where individuals carrying at least one T–T haplotype at rs1800497 and rs2283265 proposed significantly lower amounts than those not carrying this haplotype [90]. *DRD2* has long been a candidate gene for schizophrenia and other psychiatric disorders and was recently implicated in the list of genome-wide significant loci in the Psychiatric Genomics Consortium meta-analysis of schizophrenia genome-wide studies [91]. However, genomic studies on neuroeconomic gameplay are still in their infancy, with no current genome-wide study results available, and the candidate gene studies conducted suffered from small sample sizes required to detect the modest effects of most genomic variation. Further research is needed to determine whether genes or biological pathways associated with different aspects of social dysfunctional decision-making can be identified and therefore targeted for the development of treatments.

A few studies have assessed performance on neuroeconomic games before and after an intervention. Depletion of serotonin, which is implicated in social behaviour, increases rejection of unfair Ultimatum Game offers in healthy controls [92] and reduces acceptance of unfair offers in patients with GAD [47], so serotonin may modulate individuals’ perceived fairness of a situation. Oxytocin is involved in prosocial behaviour, but there is no clear impact of oxytocin administration on healthy controls playing the Trust Game [93]. Finally, disrupting neuronal activity in the right dlPFC using repetitive transcranial magnetic stimulation reduced rejection of unfair offers in an Ultimatum Game [94] and made people more likely to defect on a Trust Game, suggesting that this region is involved in overriding selfish interests and the ability to maintain a positive reputation [95]. The fact that behaviour can be altered by these interventions suggests the possibility of identifying molecular or neural mechanisms that could be used therapeutically, but again, more work is required to determine exactly which aspects of behaviour could be targeted.

Neuroeconomic games could therefore be used in future to stratify patients into diagnosis-spanning subgroups based on their social decision-making ability, or to identify targets for behavioural, pharmacological or genetic interventions to alleviate the impact of dysfunctional social decision-making in psychiatric disorders.
